# Genetic and Clinical Characteristics of *ARID1A* Mutated Melanoma Reveal High Tumor Mutational Load without Implications on Patient Survival

**DOI:** 10.3390/cancers14092090

**Published:** 2022-04-22

**Authors:** Carl Maximilian Thielmann, Johanna Matull, Sebastian Roth, Jan-Malte Placke, Eleftheria Chorti, Anne Zaremba, Georg Lodde, Philipp Jansen, Frederik Krefting, Julia Kretz, Inga Möller, Antje Sucker, Annette Paschen, Elisabeth Livingstone, Lisa Zimmer, Selma Ugurel, Dirk Schadendorf, Eva Hadaschik, Klaus G. Griewank

**Affiliations:** 1 Department of Dermatology, University Hospital Essen, University of Duisburg-Essen, Hufelandstr. 55, 45147 Essen, Germany; carlmaximilian.thielmann@uk-essen.de (C.M.T.); johanna.matull@uk-essen.de (J.M.); jan-malte.placke@uk-essen.de (J.-M.P.); eleftheria.chorti@uk-essen.de (E.C.); anne.zaremba@uk-essen.de (A.Z.); georg.lodde@uk-essen.de (G.L.); philipp.jansen@uk-essen.de (P.J.); frederik.krefting@uk-essen.de (F.K.); julia.kretz@uk-essen.de (J.K.); inga.moeller@uk-essen.de (I.M.); antje.sucker@uk-essen.de (A.S.); annette.paschen@uk-essen.de (A.P.); elisabeth.livingstone@uk-essen.de (E.L.); lisa.zimmer@uk-essen.de (L.Z.); selma.ugurel@uk-essen.de (S.U.); dirk.schadendorf@uk-essen.de (D.S.); eva.hadaschik@uk-essen.de (E.H.); 2 German Cancer Consortium (DKTK), Partner Site Essen, 45147 Essen, Germany; 3 Department of Anesthesiology, Medical Faculty, University Hospital Duesseldorf, Heinrich-Heine-University Duesseldorf, Moorenstr. 5, 40225 Duesseldorf, Germany; sebastian.roth@med.uni-duesseldorf.de; 4 Department of Dermatology, University Hospital Bonn, Venusberg-Campus 1, 53127 Bonn, Germany

**Keywords:** *ARID1A*, melanoma, mutation profiling

## Abstract

**Simple Summary:**

Melanoma is a highly malignant skin cancer with the highest mortality of all cutaneous tumors. Relevant genetic events have been identified, which shape the tumor and also the response to treatment. Recurrent *ARID1A* mutations have been identified, which are associated with improved outcomes to immune checkpoint inhibition in various tumors. Not much was known about the role of *ARID1A* mutations in melanoma to date. We investigated the largest cohort of *ARID1A* mutated melanoma to date and were able to show that despite a high mutational load the described beneficial treatment response is not apparent in melanoma.

**Abstract:**

(1) Background: Melanoma has the highest mortality of all cutaneous tumors, despite recent treatment advances. Many relevant genetic events have been identified in the last decade, including recurrent *ARID1A* mutations, which in various tumors have been associated with improved outcomes to immunotherapy. (2) Methods: Retrospective analysis of 116 melanoma samples harboring *ARID1A* mutations. Assessment of clinical and genetic characteristics was performed as well as correlations with treatment outcome applying Kaplan–Meier (log-rank test), Fisher’s exact and Chi-squared tests. (3) Results: The majority of *ARID1A* mutations were in cutaneous and occult melanoma. *ARID1A* mutated samples had a higher number of mutations than *ARID1A* wild-type samples and harbored UV-mutations. A male predominance was observed. Many samples also harbored *NF1* mutations. No apparent differences were noted between samples harboring genetically inactivating (frame-shift or nonsense) mutations and samples with other mutations. No differences in survival or response to immunotherapy of patients with *ARID1A* mutant melanoma were observed. (4) Conclusions: *ARID1A* mutations primarily occur in cutaneous melanomas with a higher mutation burden. In contrast to findings in other tumors, our data does not support *ARID1A* mutations being a biomarker of favorable response to immunotherapies in melanoma. Larger prospective studies would still be warranted.

## 1. Introduction

Melanoma is a skin tumor that, if metastasized, has a poor prognosis. Despite considerable advances in terms of overall survival made in recent years with the introduction of anti-PD-1 and anti-CTLA4 immunotherapies (ICI) or BRAF/MEK targeted therapies (TT) the 5-year survival rate remains less than 60%. The best outcomes have been achieved with the combination of the anti-PD-1 antibody nivolumab and anti-CTLA4 antibody ipilimumab having a median overall survival (OS) of over 70 months in the most recently published 6.5-year data from the Check Mate 067 study. This is, however, associated with a high rate of side effects and toxicity [1,2].

The broad availability of next-generation sequencing (NGS) technologies has enabled oncologists and scientists alike to gather a deepened understanding of genetic alterations responsible for tumor formation and potential therapeutic implications. Tumor sequencing (mostly NGS) has become a diagnostic standard, as some tumor-specific genetic alterations allow the use of novel patient- and tumor-specific therapies. Classifying melanoma by genetic alterations remains controversial [3,4]. The most common activating genetic alterations in melanoma include v-Raf murine sarcoma viral oncogene homolog B (*BRAF)* (~50%), RAS viral oncogene homolog (*RAS)* (~25%) and Neurofibromin 1 (*NF1)* (~15%) mutations. The Cancer Genome Atlas (TCGA), which performed a large whole exome analysis of patients with primary or metastatic melanoma, has suggested classifying into four main subtypes: *BRAF* mutated, *NRAS* mutated, *NF1* mutated, or triple wild-type [5]. The most commonly activated pathway via mutations in the tumor is the mitogen-activated protein kinase (MAPK) pathway. This activation mostly occurs in the previously mentioned genes, through activating mutations in the V600 codon of *BRAF* or Q61, G12 or G13 codons of *RAS* genes, or through inactivating mutations in the *NF1* gene [5].

Other mutations in melanoma are common largely due to a high number of UV-induced mutations which is why melanoma has one of the highest tumor mutational burdens (TMB) of any major cancer entity [6]. There are, however, considerable differences in mutation frequency and patterns in regard to the origin of the primary tumor (e.g., mucosal, cutaneous, uveal) [7,8,9,10]. AT-Rich Interaction Domain 1A (*ARID1A)* is one of the genes most commonly mutated in melanoma without presenting a mutation hotspot (i.e., V600 in *BRAF*). *ARID1A* encodes for the AT-Rich Interactive Domain-containing protein 1A. It is well-known as a member of the switching/sucrose nonfermentable (SWI/SNF) complex, which plays a critical role in chromatin remodeling and tumor epigenetics [11]. *ARID1A* mutations are detected with high frequency in tumor samples of entities including endometrioid and clear-cell ovarian cancer (>40%), gastric cancer, bladder cancer, hepatocellular cancer, colorectal cancer and melanoma (11.5%) [12]. *ARID1A* mutations often lead to its inactivation and subsequently to the loss of the associated protein [13,14]. Deficiency of ARID1A has been described to be associated with an increased programmed cell death-ligand 1 (PD-L1) expression, a high tumor mutational burden (TMB), impaired mismatch repair (MMR) and a modulated tumor microenvironment. Based on these findings an improved response to immune-checkpoint inhibitors has been proposed [15,16,17]. However, the clinical significance of *ARID1A* mutations, especially in melanoma, remains undefined due to a lack of studies.

In the presented study, we retrospectively assessed a large cohort of targeted next generation sequenced melanoma from 2013–2020 and were able to identify the largest cohort of *ARID1A* mutated melanomas to date. The aim was to better characterize the role of *ARID1A* mutations in melanoma, in particular, with regard to its clinical and therapeutic relevance.

## 2. Materials and Methods

### 2.1. Patients and Clinical Samples

Screening 3837 NGS reports for patients with melanoma diagnosed between 2013 and 2020 at the Department of Dermatology, University Hospital Essen, 242 patients with ARID1A mutated melanoma were identified. Related clinical data and tumor samples were available for *n* = 116 patients and were obtained from the Westdeutsche Biobank Essen, University Hospital Essen. Data for *n* = 126 patients were unavailable as tissue samples were sequenced at the University Hospital Essen, but relevant corresponding clinical, follow-up and treatment data were not available. Data for *n* = 126 patients were unavailable as tissue samples were sequenced at University Hospital Essen, but did not receive follow-up or treatment, or were included in clinical trials. Tumors were classified as per the American Joint Committee on Cancer (AJCC 8th) staging system [18]. This study was approved by the Ethics Committee of the Medical Faculty of the University of Duisburg-Essen (ethics approval no. 21-9839-BO) and followed the guidelines for good clinical practice. This study was performed in accordance with the Declaration of Helsinki and all patients gave written informed consent to be registered in the local biobank.

### 2.2. DNA Isolation

FFPE tissue was prepared according to the following protocol: 10 μm sections were first deparaffinized according to a widely known standardized procedure, consisting of two steps of 5 min xylene, 5 min 100% ethanol, 5 min 95% ethanol, 5 min 70% ethanol, 5 min 50% ethanol, followed by a rinse in water. After this process samples were air dried and tissue was then macrodissected manually for further preparation. The Genomic DNA was later isolated by using an isolation kit of Qiagen (QIAamp DNA Mini Kit (Qiagen, Hilden, Germany) Iand the manufacturer’s instructions were followed.

### 2.3. Targeted Sequencing

An amplicon based sequencing panel was customized to cover 29 genes, which are well-known to be mutated in melanoma and to cover the TERT promoter region (list of genes: Table S2, supplemental data S1). Sequencing data were analyzed by applying the CLC Cancer Research Workbench from QIAGEN (currently version 20.0.4). The CLC workflow included adapter trimming as well as read pair merging before human reference genome (hg 19) mapping. InDels and structural variants were assessed and allowed three maximum mismatches (unaligned end breakpoints). Single nucleotide variant (SNV) detection, realignment, and primer trimming were assessed afterward. Potential mutation type information, known single nucleotide polymorphisms and conservation scores by cross-referencing varying databases (COSMIC, ClinVar, dbSNP, HAPMAP, 1000 Genomes Project, and PhastCons-Conservation_scores_hg19) were obtained. After performing the previously described CLC Cancer Research workbench, a manual analysis of the data was followed. Mutations in the protein-coding portion of the gene were considered if predicted to result in non-synonymous amino acid changes. Prediction of functional implications of mutations was performed later through an analysis of server-based SIFT, PROVEAN, and PolyPhen-2 assays. A list detailing all mutations detected with the corresponding database references is shown in. In order to eliminate questionable background mutation calls (low frequency), mutations were only reported if ≥10 reads reported the mutated variant, coverage of the e mutation site was ≥30 reads and frequency of mutated reads was ≥10%. The average read coverage of the targeted area achieved in the study was 2437×.

### 2.4. Statistical Analysis

Associations of clinical parameters and tumor origin were investigated using chi-squared tests and Fisher’s exact tests, as statistically indicated. All continuous variables are depicted as means with standard deviation or as median with interquartile range, as appropriate. Categorical variables in this study are shown as total counts and percentages. All survival curves obtained in this study were analyzed using the Kaplan–Meier method with log-rank testing for all comparisons between the groups. Overall survival was calculated from the first date of stage IV diagnosis or the start of ICI/TT therapy until death or last patient contact (censored observation), respectively. Progression free survival was calculated from the start of therapy until progression or death, whichever occurs first. Statistical analyses were performed using GraphPad Prism (version 6), Microsoft Excel, SPSS 27.0 (IBM Corp., Armonk, NY, USA), R (R version 4.0.3 (10 October 2020)) and RStudio [19,20]. A *p*-value < 0.05 was considered significant.

## 3. Results

### 3.1. Patient Characteristics

One hundred sixteen patients diagnosed with melanoma were included in this cohort study (Table 1), 40 patients were female and 76 were male. The median age at first diagnosis of all patients was 61 years with an interquartile range from 22 to 94 years. At first diagnosis, 54 (46.6%) patients were younger than 60 years of age, whereas 62 (53.4%) were older than 60 years of age. In 97 (83.6%) cases the origin of the primary tumor was cutaneous, in three (2.6%) cases the melanoma was of mucosal origin, and 16 (13.8%) cases were of occult origin. Of all cutaneous cases, the most commonly reported location was the trunk (36 cases, 37.1%), followed by the head and neck region (27 cases, 27.8%), lower extremity (26 cases, 26.8%) and upper extremity (eight cases, 8.2%). *BRAF* V600E mutations were present in 61 samples (52.6%). Activating mutations in *RAS* genes were somewhat less common with mutations in 40 samples (35%). Activating *NRAS* mutations were detected in 36 samples (31%, 1 G12A, 1 G12S, 15 Q61K, 10 Q61L and 9 Q61R mutations), further three activating *KRAS* mutations (1 G12D, 1 G12A, 1G12V) and one activating *HRAS* (G13D) mutation were present. *NF1* mutations were present in 48 samples (41.4%), respectively. Mutations in *ARID1A* were reported in all 116 samples.

### 3.2. ARID1A Mutated Melanoma Harbors More Mutations Compared to ARID1A-wt Melanoma

**Figure 1:**
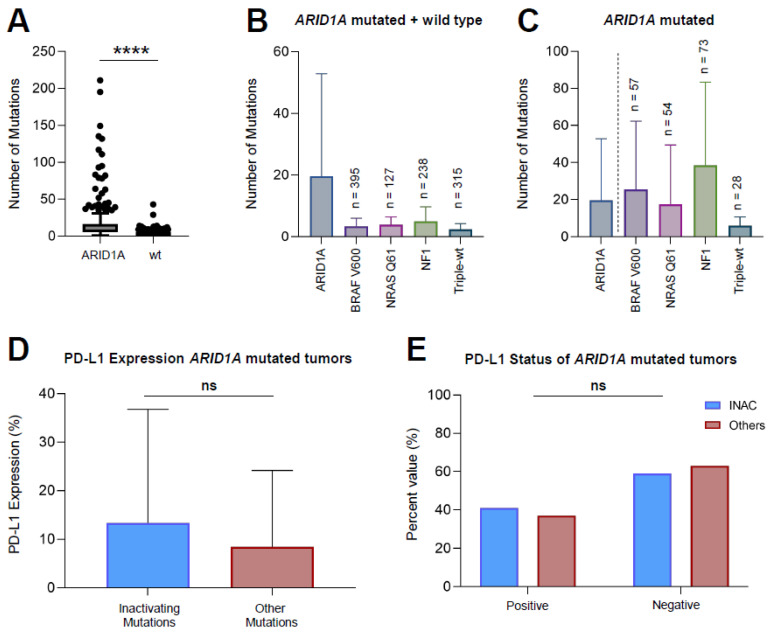
Mutation analysis in ARID1A mutated melanoma. *ARID1A* mutated melanoma harbored more mutations compared to *ARID1A*-wt melanoma (**A**). *ARID1A* mutated melanomas exhibit a higher mutation number compared to other melanoma subtypes (**B**). Within the group of *ARID1A* mutated melanomas, NF1 mutant samples exhibit the highest number of mutations (**C**). PD-L1 expression levels did not differ between samples with inactivating and other ARID1A mutations. The rate of PD-L1 positive tumors was comparable between the groups (**D**,**E**). Statistical tests performed are Mann–Whitney U tests. Data are shown as mean ± SEM. **** *p* < 0.0001. ns: no significant; *p* < 0.0001.

An analysis of mutational patterns of *ARID1A* mutated melanomas (*n* = 116) versus *ARID1A* wild-type melanomas (*n* = 1180) revealed a significantly higher number of mutations in *ARID1A* mutated melanomas (mean 19.6 mutations versus 3.3 mutations per sample) (*p* < 0.0001) (Figure 1A). A subgroup analysis of the wild-type cohort into *BRAF* V600, *NRAS* Q61, *NF1* and Triple-WT showed that *ARID1A* mutated melanomas exhibit higher amounts of mutations compared to all other melanoma subtypes (mean number of mutations: *ARID1A*mut [19.6], *BRAF* V600mut [3.4], *NRAS* Q61mut [3.9], *NF1*mut [5.0], Triple-WT [2.4]) (Figure 1B). Within the group of *ARID1A* mutated melanomas (Figure 1C), the mutational pattern with *NF1* mutated melanomas harboring the greatest mutational load. Male patients had a higher mutational load (mean = 26.97 mutations per sample) compared to female patients (mean = 15.67 mutations per sample), although not statistically significant (*p* = 0.061).

### 3.3. Inactivating Mutations of ARID1A Do Not Lead to a Greater PD-L1 Expression Compared to Other Mutations

The analysis of the PD-L1 expression revealed no significant difference in expression levels of *ARID1A* mutated tumors with inactivating frameshift or nonsense mutations (INAC) compared to others (mean 13.3 versus 8.4, respectively; *p* = 0.3060) (Figure 1D). Further, the rate of PD-L1 positive (>5% PD-L1 expression) tumors was comparable among both INAC and samples exhibiting other mutations (Figure 1E). Within the INAC group (*n* = 32), nine samples (28.1%) were PD-L1 positive, 13 samples (40.6%) were negative, and 10 samples (31.3%) were not tested. Of all other samples (*n* = 84), 23 samples (27.4%) were PD-L1 positive, 40 samples (47.6%) were negative, and 21 (25.0%) were not tested. This distribution did not show any statistical significance (Table 2).

### 3.4. Survival Analysis of ARID1A Mutated Malignant Melanoma

**Figure 2:**
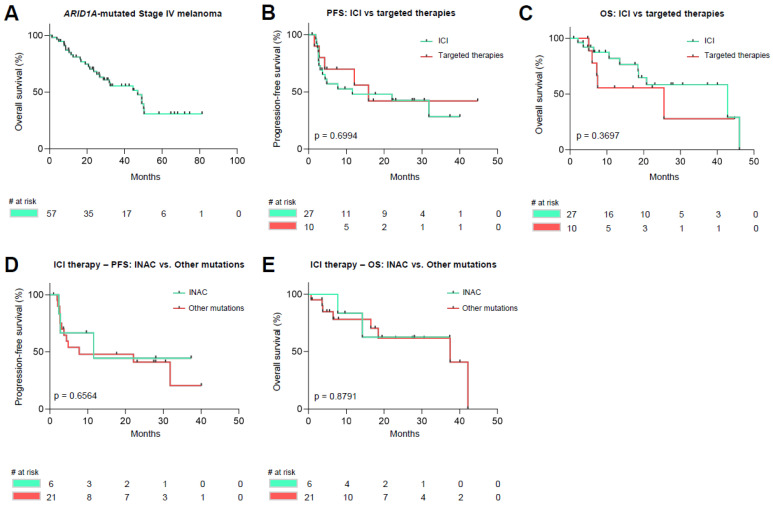
Survival Analysis of *ARID1A* mutated melanoma. Overall survival of *ARID1A* mutated stage IV melanoma (**A**). No difference in progression-free or overall survival was noticed comparing patients who received either immune checkpoint inhibitors or targeted therapies as their first-line non-adjuvant therapies (**B**,**C**). Patients with inactivating *ARID1A* mutations did not differ in progression-free and overall survival compared to other mutations (**D**,**E**).

Survival analysis revealed a median overall survival (OS, calculated from date of stage IV diagnosis) of 47.6 months for all Stage IV *ARID1A* mutated samples (*n* = 57) included in this study (Figure 2A). Upon further analysis, *ARID1A* mutated melanoma patients receiving their first non-adjuvant systemic therapy (with either targeted therapies or immune-checkpoint inhibitors) (*n* = 37). No statistical significance was noticed upon comparing patients receiving targeted therapies or therapies with immune-checkpoint-inhibitors in both progression-free and overall survival (median PFS (mPFS) ICI-cohort [*n* = 27]: 11.6 months versus mPFS TT-cohort [*n* = 10]: 15.9 months, *p* = 0.6994; median OS (mOS) ICI-cohort [*n* = 27]: 42.8 months versus mOS TT-cohort [*n* = 10]: 25.5 months, *p* = 0.3697) (Figure 2B,C). No difference in terms of progression-free- and overall survival was noticed upon comparison of INAC (*n* = 6) versus other mutated samples (*n* = 21) upon receiving therapy with immune-checkpoint inhibitors (mPFS INAC: 11.6 months versus mPFS others: 7.8 months, *p* = 0.6564; mOS INAC: not met versus mOS others: 37.5 months, *p* = 0.8791) (Figure 2D,E).

### 3.5. Distribution of UV-Induced Mutations amongst Melanoma Samples

An analysis of mutational patterns within the *ARID1A* mutated tumor samples revealed UV-induced signature mutations (Figures S1 and S2). Apparent single nucleotide variants were classified according to six different mutation types, as previous studies have conducted before [21]. *ARID1A* mutated melanomas harbor the greatest amount of C>T substitutions at the dipyrimidine upon comparison with Triple-WT melanoma in both absolute and relative numbers (Figure S1). The same signature was noticeable when looking at UV-induced CC>TT substitutions, in which the greatest amount was noticed among the *ARID1A* mutated melanoma subtype upon comparison with Triple-WT melanoma (Figure S2).

### 3.6. Targeted Next Generation Sequencing of ARID1A Mutated Melanoma

**Figure 3:**
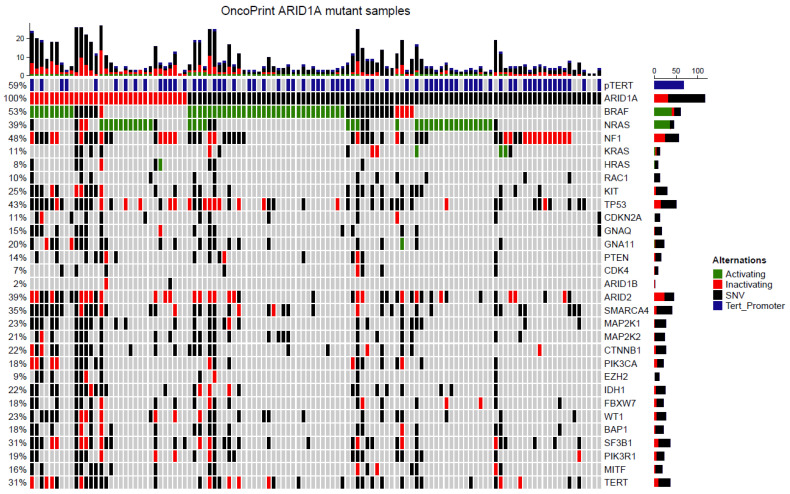
Oncoplot of *ARID1A* mutated melanoma. Mutation distribution in *ARID1A* mutated melanoma. Green: mutations known or assumed to be activating. Red: loss of function mutations. Blue: known activating mutations in the TERT promoter region.

**Figure 4:**
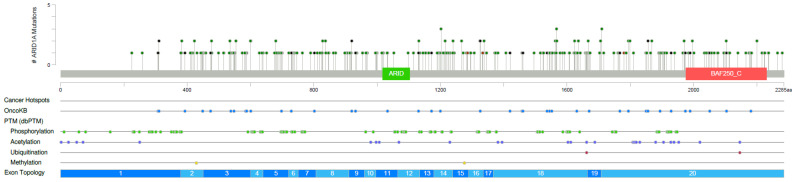
Mutation distribution in ARID1A. Lollipop mutation graph demonstrating the distribution of mutations. Missense mutations are demonstrated in green, inactivating (Nonsense or frame-shift mutations) in black, in frame frameshift mutations in brown.

Mutations were identified in all 116 samples (Figure 3, Table S1) included in this study. In these samples in total, 297 *ARID1A* mutations were identified, with many samples harboring more than one mutation. *ARID1A* mutations were distributed evenly without clustering or a hotspot (Figure 4). The most frequently mutated gene in addition to *ARID1A* was *BRAF* (*n* = 61, 52.6%). Of all *BRAF* mutations, 38 samples had activating V600E mutations, three samples had activating V600K mutations, and one sample had a V600D activating mutation (Table S1). *NRAS* mutations were found in 45 samples (38.8%), of which 36 were activating Q61/G12/G13 mutations (Table S1). Mutations in *KRAS* and *HRAS* were detected less frequently with three (1 G12D, 1 G12A, 1G12V) and one (G13D) activating mutations, respectively (Figure 3). *NF1* mutations were present in 56 samples (48.3%). Activating TERT-promoter mutations were present in 68 samples (58.6%) (Table S1, Figure 3). Other frequently mutated genes included *TP53* (43%), *ARID2* (39%), and *SMARCA4* (35%). Interestingly, only two samples harbored an *ARID1B* mutation. Other less frequent mutations were reported in various genes including *CDKN2A*, *GNAQ*, *GNA11*, *PTEN*, *CDK4*, *MAP2K1*, *MAP2K2*, *CTNNB1*, *PIK3CA*, *EZH2*, *FBXW7*, *IDH1*, *WT1*, *BAP1*, *RAC1*, *SF3B1*, *PIK3R1*, *MITF*, and TERT.

## 4. Discussion

In this study, we screened 3837 reports from next generation sequencing between 2013 and 2020 for *ARID1A* mutated melanoma and were able to identify 116 individual patients with available clinical data harboring an *ARID1A* mutation. Due to the relatively large size of the cohort, we were able to further distinguish between the subgroup of tumors with inactivating *ARID1A* mutations and others. To our knowledge, this is the largest cohort of *ARID1A* mutated melanoma investigated to date.

Interestingly, in our cohort *ARID1A* mutations appeared almost exclusively in cutaneous melanoma. Mutations in the *ARID1A* gene were otherwise found in three samples (2.6%) of patients harboring mucosal melanomas. The remaining samples in which *ARID1A* mutations were detected were melanomas without known primary origin. However, genetic evidence argues these tumors mostly originate from cutaneous sites, as they demonstrate a similar distribution of *BRAF*, *NRAS* and *NF1* mutations to cutaneous melanoma [22]. The clinical origin and genetic data of *ARID1A* mutant melanoma argue that these tumors arise almost exclusively in UV-exposed sites and are rare in non-UV-exposed subtypes including mucosal, acral and uveal melanomas. Further, *ARID1A* mutated melanomas predominantly appear in the male population, making up almost two-thirds of all affected patients. Large analyses of cutaneous melanomas regardless of the mutational status did not reveal a similar distribution pattern [23,24]. The reason for the observed male predominance in our study of *ARID1A* mutated melanoma is currently not apparent to us.

Within the group of *ARID1A* mutated melanomas, higher numbers of accompanying mutations were noticed compared to *ARID1A* WT cases. The most common mutation found was a C>T substitution. Both findings go together with previously published data on *ARID1A* mutated tumors, including ovarian cancer, hepatocellular carcinoma, colorectal adenocarcinoma, and non-small-cell lung cancer (NSCLC) [15,16,25,26]. Investigation of subgroups upon their mutational profile showed that the tumor mutational load within the group of *ARID1A* mutated tumors is dependent on its co-mutations. The described pattern of *NF1* mutant melanoma having the largest number of mutations within the three main subtypes of *BRAF*, *NRAS*, or *NF1*-mutated melanomas was apparent in our *ARID1A* mutated cohort [27]. Although not statistically significant, our cohort observed a clear trend towards a higher mutational load in male patients, which goes in line with previously published data [28]. A large tumor mutational burden has been linked to improved responses to immune-checkpoint inhibitors. This would suggest that *ARID1A* mutated tumors might show a better response to immune-checkpoint inhibitors [29,30]. Recently, a high TMB of ≥10 mut/Mb has been approved as a cut-off to select patients for therapy with anti-PD-1 agent Pembrolizumab. Considering our observed differences in mutational load with regard to sex and previous findings, sex specific TMB cut-offs may be something that should be considered.

Further, it has been described that *ARID1A* deficient tumors have a higher level of PD-L1 expression compared to WT-correlates in a variety of different cancer subtypes [17,31]. A higher PD-L1 expression has been linked to an improved response to immune checkpoint inhibitors in various cancers, including melanoma [32,33,34]. Hence, we have investigated the PD-L1 expression within the *ARID1A* mutated tumors grouped into inactivating and other mutations. This analysis did not reveal a significantly higher rate of PD-L1 expression among tumors with inactivating *ARID1A* mutations. Further, the rate of PD-L1 positivity was comparable to previously described melanoma cohorts with roughly 40% of samples showing a PD-L1 expression greater than 5% [33,35]. Upon comparison of patients with either inactivating or other mutations within the investigated *ARID1A* cohort, we were unable to recognize a difference in survival rates. This may be due to a similar tumor mutational burden and similar rates of PD-L1 expression.

Survival analysis of stage IV melanoma patients harboring *ARID1A* mutations and comparisons with previously published data did not reveal a difference in terms of overall survival [27]. In addition, we did not notice a difference in terms of overall- or progression-free survival depending on both therapeutic regimen (targeted therapies vs. immune-checkpoint inhibitors) or type of *ARID1A* mutation (inactivating vs. others). No survival advantage of *ARID1A* mutated melanoma is apparent comparing survival data with the most recently published data on ICI in the CheckMate 067 study [36]. This argues against previous findings, in which *ARID1A* mutated tumors were thought to exhibit a better response to immune-checkpoint inhibitors [15,16]. *ARID1A* deficiency has been related to a compromised mismatch repair pathway, expression of programmed cell death ligand 1 (PD-L1) and tumor mutational burden [17]. A possible reason we were unable to notice the believed effects may be the fact that Okamura et al. have investigated an inhomogeneous cohort of nine cancers, of which 375 did receive immune-checkpoint inhibitors as a therapeutic regimen and cancer-specific differences in terms of therapeutic responses and survival were not individually assessed. We believe if a strong benefit of *ARID1A* mutant samples to immune-checkpoint inhibitors was present in melanoma, our study would have detected it.

Our data show that patients with *ARID1A* mutated melanoma treated with immunotherapy exhibit no better overall survival than those with *ARID1A*-wild-type melanoma and within the *ARID1A* mutated cohort, no differences between inactivating and other *ARID1A* mutations in terms of both overall- and progression-free survival were apparent. This finding suggests *ARID1A* mutations have no large impact on survival and especially immune-checkpoint inhibitors as has been suggested, at least for melanoma. This argues determining *ARID1A* mutation status in melanoma is currently not relevant for treatment. However, our study lacks the prospective aspect of clinical studies but goes in line with data from Alaiwi et al. [37]. Ideally prospectively collected larger datasets should be analyzed to further assess the ideal therapeutic regimen and possible further implications of *ARID1A* mutations on metastatic melanoma.

## 5. Conclusions

*ARID1A* mutations primarily occur in cutaneous melanomas with a UV-signature high mutation burden. Larger prospective studies are warranted, however, our data assessing the largest cohort of *ARID1A* mutated melanoma presented to date does not support *ARID1A* mutations being a biomarker of response to immunotherapies in melanoma.

## Figures and Tables

**Table 1:** Clinical characteristics of *ARID1A* mutated melanomas (*n* = 116).

Variable, n (%)	
**Age**	
Median	61
Range	22–94
≤60	54 (46.6)
>60	62 (53.4)
**Sex**	
Female	40 (34.5)
Male	76 (65.5)
**Mutated Oncogene**	
BRAF V600E	61 (52.6)
NRAS Q61	45 (38.8)
NF1	48 (41.4)
ARID1A	116 (100)
**Primary Tumor**	
Cutaneous	97 (83.6)
Mucosal	3 (2.6)
Occult	16 (13.8)
**Subtype of Cutaneous Tumors**	
SSM	25 (21.6)
NMM	35 (30.2)
ALM	11 (9.5)
LMM	2 (1.7)
Desmoplastic	4 (3.4)
Spitzoid	2 (1.7)
Unknown	18 (15.5)
**Ulceration**	
Present	49 (42.2)
Missing	37 (31.9)
Unknown	30 (25.9)
**Sentinel Lymph Node Biopsy**	
Positive	24 (20.7)
Negative	44 (37.9)
Not performed	48 (41.4)
**PD-L1**	
Positive	31 (26.7)
Negative	56 (48.3)
Unknown	29 (25.0)
**Tumor Thickness**	
<1 mm	9 (7.8)
1–2 mm	24 (20.7)
2–4 mm	27 (23.3)
>4 mm	31 (26.7)
Unknown	24 (20.7)
**Tumor Location**	
Trunk	36 (37.1)
Lower Extremity	26 (26.8)
Upper Extremity	8 (8.2)
Head and Neck	27 (27.8)

**Table 2:** PD-L1 Expression of ARID1A mutated tumors.

Variable (*n*, %)	INAC (*n* = 32)	Others (*n* = 84)	*p*-Value
PD-L1 positive (>5%)	9 (28.1)	23 (27.4)	0.71
PD-L1 negative (<5%)	13 (40.6)	40 (47.6)	
Not tested	10 (31.3)	21 (25.0)	

## Data Availability

Not applicable.

## Supplementary Materials

The following supporting information can be downloaded at: https://www.mdpi.com/article/10.3390/cancers14092090/s1, Figure S1: *ARID1A* mutated melanoma harbor more C>T substitutions than *ARID1A*-wild type melanoma; Figure S2: *ARID1A* mutated melanoma harbor more CC>TT UV-induced dinucleotide substitutions compared to *ARID1A*-wild type melanoma; Table S1: Mutational overview of BRAF, NRAS, NF1, ARID1A, TERT promoter; Table S2: Genes covered in the applied sequencing panel; Table S3: Clinical characteristics of patients receiving non-adjuvant systemic therapy; Supplementary data S1: Mutational data of all patients
