# Supplementary material for: Genetic and Clinical Characteristics of ARID1A Mutated Melanoma Reveal High Tumor Mutational Load without Implications on Patient Survival
Source: Cancers (Basel). 2022 Apr 22;14(9):2090. doi: 10.3390/cancers14092090 (PMC9101535; doi:10.3390/cancers14092090)
Supplement: Supplementary file 1 [file cancers-14-02090-s001.zip › cancers-1666969 Supplementary material.pdf]

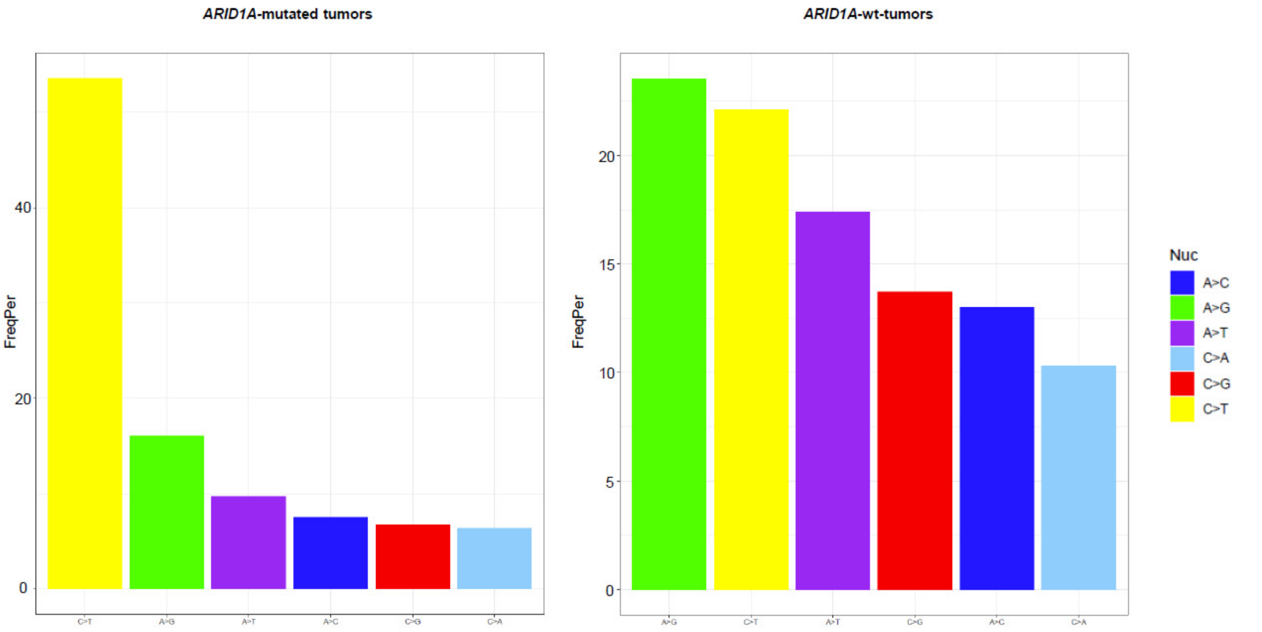

Figure S1. *ARID1A* mutated melanoma harbor more C>T substitutions than *ARID1A*-wild type melanoma.

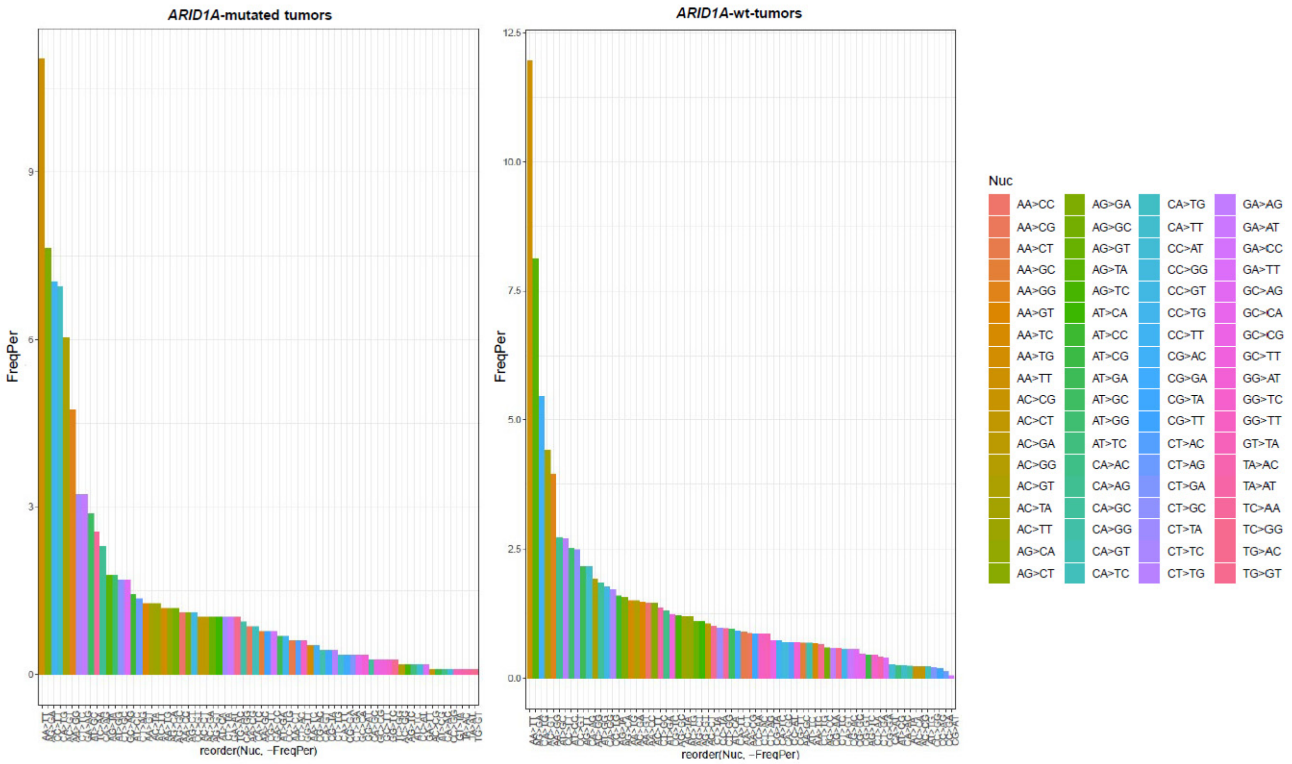

Figure S2. *ARID1A* mutated melanoma harbor more CC>TT UV-induced dinucleotide substitutions compared to *ARID1A*-wild type melanoma.

Table S1. Mutational overview of BRAF, NRAS, NF1, ARID1A, TERT promoter.

BRAF	*NRAS*	*NF1*	*ARID1A*	TERT Promoter
V600K; D66N		G2052E; M1285I	P848L; S446F	1295228
V600K	Q61K	P2242S	S1153F	
V600K			F2103V	1295228
V600E; V600K	D33E	W2229*; W425*	G1849E; G410R	
V600E; S314F		D2005G; W1685*	A837T; M828I	
V600E; R69K		E2211K; L2116F	M628I; Q1172*	
V600E; R50K		L2604F; V1677I	G1267R; H1624Y	
V600E; R165Q	S65N; V188M	D1485N; S2460L	M1224I; P582S	1295250
V600E; L144S	Q61R	F1289S; S1355L	G1282R; K1382R	1295228
V600E; G85D		G849R; S1566N	D1231Y; G1996D	
V600E; G219E	R123G	K1263E; K2823E	M1215I; S539L	1295250
V600E; A133V	Q61L	R2349H; R752K	P1633S; P690L	1295250
V600E			D1193G	
V600E			H1624Y; P1132S	
V600E			P1966L	
V600E			P1567S	
V600E			N906K	
V600E		V172I	D1286_R1287delinsC*; Y1285S	
V600E			G2040R	
V600E			R2236H	
V600E		I1186T; S2543N	M1578I; P225S	
V600E		Q1255*; S1500N	A1622T; T1198I	
V600E			P1710S; Q1856*	
V600E		M1376V	R1202Q	
V600E	Q61K		G1375S	1295250
V600E		G72E	S258F	1295250
V600E			I1907F	1295250
V600E			R2183C	1295250
V600E			M828I; P1568S	1295250
V600E			P459S	1295250
V600E			P680L	1295250
V600E			E1033*; E1033*	1295250
V600E			S862F	1295228
V600E			L1607Q	1295228
V600E		E244G; P2242L	P380S	1295228
V600E			P1563S	1295228
V600E			S735N	1295228
V600E			R1053C	1295228
V600E			R1020W	1295228
V600E			D1850fs; S1929fs	1295228
V600E			L1716F	1295228
V600D			R1906Q	1295228
V377I		D832N; V253M	G1665E; R1566K	
S209_R210delins*; V600fs			L2112F	1295250
S16L	Q61K; R97K	V497M	P1567L; P819L	1295228
R278Q	G177E	G2811R; V2572I	S696F; S936N	
R278*	Q61L	A2333V; Q2636R	P1372L	
Q98*			P636L; S2179N	

P75L		A1294T; A2333T	G827D; W1545*	1295228
P367S	Q61L		S536F	1295250
P334Q			Q1145K	
P29S	E132K	P464L; V530I	P1326fs; P1407S	
P239L; S363F		L2620F	S574F	1295228
P229S; P321S	A66V; G115R	A1294V; E1571K	A905T; P600L	
N140S		P2758S; T1950I	G443S; Q1974*	
L313F; V281I	Q61R	A456T; H2658Y	Q449*; R2057W	
L286F; S136L	Q61R; Y157*	A1177V; P2696L	G838R; Q920*	
G73E			P545L	
E140K	G12A	P458L; W1512*	P535L; P554S	
D201N	P34L	V1182D	P1710S	1295228
D172E; L204R			L1915F	1295228
	V14I	A132T; L121F	D2051fs; R1989*	
	Q61R	A152V	P2095L; Q1631*	
	Q61R		P383S	
	Q61K		Q1131*	
	Q61K		P418S	
	Q61K		P2102L; S2104fs	
	Q61K	V1308L	P1562L	
	Q61K		Q588*; R1202Q	
	Q150P; Q61R	P2792L	Q309P; Y311fs	
	G12S		E1767*	
	A130T	E1929K; I183T	Q1098R	
			P2140L	
			G1015S; P1642S	
			P1326fs	
			V1526M	
			P739S	
		M2526I	H477Y; S825N	
		D896N; T1184fs	V700A	
	Q61R		P1132A	1295250
	Q61L		P1213S; P452S	1295250
	Q61L		P1163S	1295250
	Q61L		P1240S	1295250
	Q61K		P729fs	1295250
	Q61K		Q1894*	1295250
	Q61K		D1794fs; Q1856*	1295250
		R1870L; R440*	Q1537*	1295250
		Q2636*; S2549L	Q1458*	1295250
		Q1070*	P533S	1295250
			P2225L	1295250
		Q756*	A2237P	1295250
			Q1200*; S682F	1295250
		Q1218*	E1778_Glu1780del	1295250
		K1263*	S1544P	1295250
		Q2234*; W1997*	P1667L	1295242
		R440*; V951I	S1338F	1295242
	Q61R		Q1334_R1335insGln	1295228
	Q61L		H1608Y	1295228
	Q61L		P2077L	1295228
	Q61L		P2114L	1295228
	Q61L	S2817F	S1338F	1295228
	Q61K; S87N		A2181fs	1295228
	Q61K		L1970F; S1516F	1295228
	Q61K		D1972N	1295228
	Q61K		S1868F	1295228
	L95P; Q61R	P1851S; S155G	E1787K; Q1346H	1295228

	R2812Q; Y794*	F2103L	1295228
	C454Y	M1661I; P1325L	1295228
	R1306Q; W1236*	S1868F	1295228
	Q1255*	S499L	1295228
	L300fs; Q1515*	A900T	1295228
	S1991Y	P1115S	1295228
	L161fs	R1461*	1295228
	Q853*; S2754F	P1667S	1295228
	R1241*; S1567L	P1307L; P1325F	1295228
		M1215I	1295228

Table S2. Genes covered in the applied sequencing panel.

Nr.	Gene	Chr	Start	End	Primary Melanoma type	Nr. Targets
1	*TERT* prom.	5	1295200	1295280	cutaneous	2
2	*BRAF*	7	140419127	140624564	cutaneous	95
3	*NRAS*	1	115247085	115259515	cutaneous	7
4	*HRAS*	11	532242	537287	cutaneous	8
5	*KRAS*	12	25357723	25403870	cutaneous	14
6	*KIT*	4	55524085	55606881	cutaneous	34
7	*RAC1*	7	6414126	6443608	cutaneous	9
8	*NF1*	17	29421945	29709134	cutaneous	111
9	*TP53*	17	7565097	7590863	cutaneous	18
10	*CDKN2A*	9	21967751	21995300	cutaneous	26
11	*GNAQ*	9	80331003	80646374	uveal	14
12	*GNA11*	19	3094408	3124002	uveal	14
13	*PTEN*	10	89622870	89731687	cutaneous	14
14	*CDK4*	12	58141510	58149796	cutaneous	11
15	*ARID1A*	1	27022522	27108601	cutaneous	67
16	*ARID2*	12	46123448	46301823	cutaneous	56
17	*SMARCA4*	19	11071598	11176071	cutaneous	60
18	*MAP2K1*	15	66679155	66784650	cutaneous	35
19	*MAP2K2*	19	4090319	4124126	cutaneous	35
20	*CTNNB1*	3	41236328	41301587	cutaneous	27
21	*PIK3CA*	3	178865902	178957881	cutaneous	37
22	*EZH2*	7	148504464	148581441	cutaneous	30
23	*IDH1*	2	209100951	209130798	cutaneous	15
24	*FBXW7*	4	153242410	153457253	cutaneous	33
25	*WT1*	11	32409321	32457176	cutaneous	21
26	*SF3B1*	2	198254508	198299815	uveal	48
27	*BAP1*	3	52435024	52444366	uveal	30
28	*PIK3R1*	5	67511548	67597649	cutaneous	32
29	*MITF*	3	69788586	70017488	cutaneous	31
30	*TERT*	5	1253147	1295068	cutaneous	36

Table S3. Clinical characteristics of patients receiving non-adjuvant systemic therapy.

Variable (n = 37)	
**Age**	
Median	64
Range	38 - 87
≤60	14 (37.8)
>60	23 (62.2)
**Sex**	
Female	15 (40.5)
Male	22 (59.5)
**Primary Tumor**	
Cutaneous	29 (78.4)
Mucosal	1 (2.7)
Occult	7 (18.9)
**Ulceration**	
Present	14 (37.8)
Missing	8 (21.7)

Unknown	15 (40.5)
**Sentinel Lymph Node Biopsy**	
Positive	7 (18.9)
Negative	12 (32.4)
Not performed	18 (48.6)
**PD-L1**	
Positive	10 (27.0)
Negative	21 (56.8)
Unknown	6 (16.2)
**LDH at therapy start**	
Elevated	4 (10.8)
WNL	19 (51.4)
Unknown	14 (37.8)
**Therapies**	
PD1 Mono	11 (29.7)
PD1 + CTLA4	16 (43.2)
Dabra Trame	7 (18.9)
Vemura Cobi	2 (5.4)
Enco Bine	1 (2.7)
**Metastatic organs**	
<3 Organs	25 (67.5)
≥3 Organs	12 (32.5)
